# Buzzing Away Pain: Efficacy of Buzzy® in Reducing Pain During Canakinumab Treatment for Familial Mediterranean Fever

**DOI:** 10.7759/cureus.81242

**Published:** 2025-03-26

**Authors:** Nebahat Zeynep Özaslan, Betül Öksel, Nihal Sahin, Hafize E Sönmez

**Affiliations:** 1 Department of Pediatric Rheumatology, Kocaeli University, Kocaeli, TUR

**Keywords:** buzzy®, familial mediterranean fever, pain, subcutaneous injection, treatment

## Abstract

Background: In colchicine-resistant patients with familial Mediterranean fever (FMF), alternative therapies, which are administered parenterally, become necessary. Effective pain management is essential for FMF treatment. Buzzy® (Pain Care Labs, Atlanta, GA) is specifically designed to alleviate pain during needle procedures. This innovative tool integrates a cold pack with a vibration motor, thereby enabling the simultaneous application of cold therapy, tactile stimulation, and distraction techniques. By harnessing the combined effects of cold and vibration on the skin, Buzzy® significantly reduces pain perception. This study aimed to evaluate the effectiveness of Buzzy® in mitigating pain during subcutaneous administration of canakinumab in patients with FMF who are resistant to colchicine. This study included patients with colchicine resistance who were receiving canakinumab. Pain scores were evaluated before and after applying Buzzy® using the Visual Analog Scale (VAS), Wong-Baker FACES Pain Rating Scale (FPS-R), and Children’s Fear Scale (CFS).

Results: A total of 15 patients were included in the study. Nine patients (60%) were female, and six (40%) were male. The median age of the patients was nine years (five to 18 years). The median VAS, FPS-R, and CFS scores before Buzzy® were four (0 to eight), four (0 to eight), and one (0 to four), respectively. After Buzzy® application, the median VAS, FPS-R, and CFS scores were two (0 to six), two (0 to six), and 0 (0 to three), respectively. The VAS and FPS-R scores showed a significant reduction (p=0.04 and p=0.008, respectively), indicating a notable decrease in pain. Although the CFS scores also decreased following the use of Buzzy®, this reduction was not statistically significant (p=0.526).

Conclusion: According to the gate control theory of pain, methods like Buzzy® temporarily block pain signals from reaching the central nervous system by "closing the gates." In patients requiring continuous injections, such applications may reduce the pain and anxiety they experience.

## Introduction

Familial Mediterranean fever (FMF) is an autoinflammatory hereditary disease characterized by recurrent episodes of fever and serositis. Although colchicine remains the cornerstone of FMF treatment, it is ineffective in 5% to 10% of patients [1]. Patients are considered colchicine-resistant if they experience one or more FMF attacks per month over the last six months despite receiving the maximum tolerated dose of colchicine, have three or more attacks within a four- to six-month period, exhibit elevations in two or more acute phase reactants during incomplete attacks, or show evidence of subclinical inflammation (elevated acute phase reactants) during intervals between attacks without overt symptoms [2]. In cases of colchicine resistance, alternative treatments are administered via parenteral routes. One widely employed parenteral therapy is canakinumab, a monoclonal antibody that inhibits the binding of interleukin-1 beta to its receptor. In conditions such as FMF, which involve painful episodes, a comprehensive approach to pain management is essential. The pain associated with the disease can be further exacerbated by the treatment methods themselves; thus, adjusting these interventions can help mitigate the overall discomfort.

According to the gate control theory of pain, pain management techniques can temporarily block pain signals from reaching the central nervous system by "closing the gates." This theory proposes that a modulation center in the dorsal horn of the spinal cord serves as a pathway for pain signals transmitted from the peripheral nervous system to the central nervous system. Activating nerve fibers that transmit non-noxious stimuli, such as cold or vibration, can reduce the perception of pain [3]. Unmyelinated A-delta and C-type nerve fibers transmit pain at a speed of 6-30 m/s and 0.5-2 m/s, respectively, whereas myelinated A-beta-type fibers transmit vibration stimulation at a speed of 30-70 m/s [4].

Buzzy® (Pain Care Labs, Atlanta, GA) is a device developed by pediatrician Dr. Amy Baxter to reduce pain during needle procedures [5]. This innovative bee-shaped device combines an ice pack with a vibration motor, allowing clinicians to simultaneously apply cold therapy, tactile stimulation, and distraction techniques. The vibration component of the Buzzy® device blocks A-delta fibers and stimulates A-beta fibers. The cold component, when used before the pain stimulus, stimulates the C-fibers and further blocks the A-delta fibers. The vibrations also stimulate mechanoreceptors such as Pacinian and Meissner bodies, not only in the skin and subcutaneous tissue but also in the underlying bone [3]. This treatment is believed to alleviate pain during injection.

Invasive procedures, such as venous blood sampling, venipuncture, and vaccination, cause pain and fear in children [6,7]. Buzzy®, which utilizes the effects of cold and vibration on the skin, has been shown to effectively reduce pain perception in pediatric patients [8-11]. In this study, we aimed to present the data of patients with colchicine-resistant FMF who received subcutaneous canakinumab and the effect of Buzzy® on pain and fear perception during subcutaneous injection.

## Materials and methods

Our study was conducted between January 2024 and June 2024. The study group included patients aged 0-18 years who were diagnosed with FMF at the Department of Pediatrics, Division of Rheumatology of Kocaeli University in Kocaeli, Turkey, and who received monthly subcutaneous injections of canakinumab due to colchicine resistance. The study was initiated after receiving ethical approval from the local ethics committee of Kocaeli University (approval number: GOKAEK-2024/05.32-2024/155; Date: 14.03.2024).

The Visual Analog Scale (VAS), Facial Pain Rating Scale (Wong-Baker FACES Pain Rating Scale; FPS-R), and Children's Fear Scale (CFS) were routinely administered to our patients at each injection. The VAS allows patients to rate their pain from 0 (no pain) to 10 (excruciating pain). The FPS-R is a tool used to measure pain levels in children. This scale includes six cartoon-like faces, ranging from a smiling face (0 = very happy/no pain) to a crying face (10 = worst pain). Children express the pain they are experiencing by selecting the degree of pain indicated by facial expressions. The validity and reliability of this scale in pain assessment have been demonstrated in previous studies [12]. The CFS is a valid and reliable scale developed to assess the levels of fear and anxiety in children. The proposed method includes five facial expressions, and a fear score is obtained by assigning numerical values to the facial expressions. The first face indicates no fear (0 points); the last face indicates too much fear (four points) [13].

In pediatric rheumatology practice, scales based on patient statements and physician opinions are routinely used. In our clinic, the VAS, FPS-R, and CFS are routinely assessed in each patient for parenteral administration.

Since the efficacy of Buzzy® has been demonstrated in previous studies [6, 7, 9, 11], Buzzy® has been administered before parenteral therapy applications in our clinic since January 2024.

**Figure 1:**
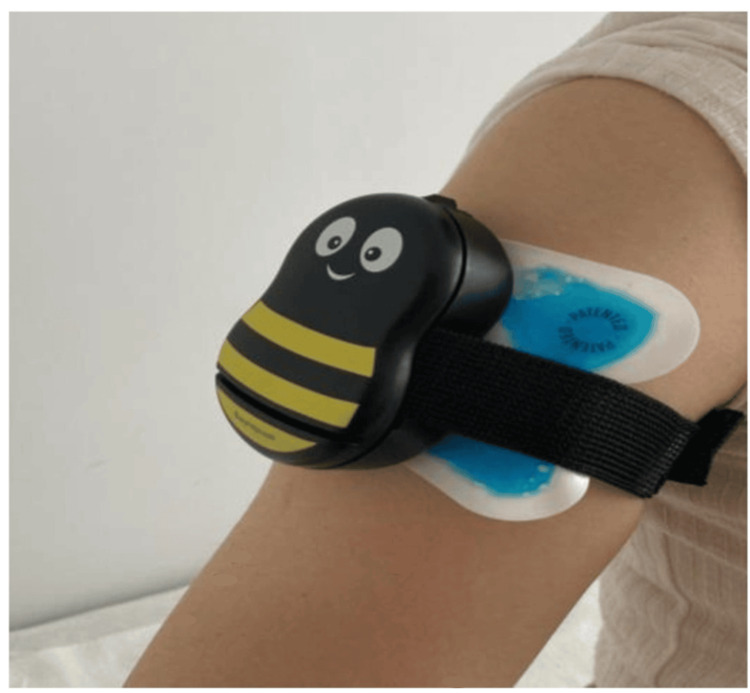
Buzzy device and its placement on the arm, i.e., the injection site.

Informed consent statements were obtained from all patients and their parents prior to subcutaneous injection and the use of the Buzzy® device. Buzzy® modulates pain perception by applying vibration and cold to the skin surface. The device reduces pain perception not by directly interacting with the brain but through pain modulation mechanisms acting on peripheral nerves. Prior to the subcutaneous injection, the child was introduced to the device, and a simple explanation of how it works was provided. The child was then given the opportunity to touch the device to become familiar with it. The cold pads of the Buzzy® device were stored in the freezer, and when the child was ready, the frozen pads were attached to the device. After placing the cold pads of the device on the appropriate dermatome, the device was activated for one minute, and the injection was administered while the vibration continued (Figure 1). After the injection, the device was removed, and once the procedure is completed, the wings of the Buzzy® device were wiped with alcohol and cooled again in the freezer.

Injection procedures were performed by the same healthcare personnel in the pediatric rheumatology outpatient clinic. In this study, the VAS, FPS-R, and CFS scores routinely recorded during interventional procedures were retrospectively reviewed from patient files.

Statistical analysis

IBM SPSS Statistics software, version 21 (IBM Corp., Armonk, NY) database was created for statistical analysis. The variables were investigated using visual (histogram, probability plots) and analytic methods (Kolmogorov-Smirnov/Shapiro-Wilk’s test) to determine whether they were normally distributed or not. In descriptive analyses, the median (minimum-maximum) value was used for non-normally distributed parameters. Categorical variables were expressed as percentages. Variables between dependent groups were compared using the Wilcoxon test.

## Results

The study included 15 patients with FMF and colchicine resistance who were receiving subcutaneous canakinumab. Of the patients, nine (60%) were female and six (40%) were male. The median age of patients was nine years (range five to 18 years). The median age at diagnosis was 5.5 years (range two to 11 years). The median duration of colchicine treatment was three years (range one to 14 years), and the median duration of canakinumab treatment was 12 months (range six to 36 months). Except for one (6%) patient with epilepsy, none of the patients had any additional diseases. The rate of consanguineous marriage was 6%, and five patients (33%) had a family history of FMF.

Upon examining the clinical findings during the attacks, 14 patients (93%) had fever, 13 (86%) had arthralgia, 11 (73%) had abdominal pain, five (33%) had myalgia, four (26%) had chest pain, four (26%) had rash, three (20%) had arthritis, three (20%) had diarrhea, three (20%) had exercise-related leg pain, one (6%) had pericarditis, and one patient (6%) had erysipelas-like rash. No patient had a history of prolonged febrile myalgia, orchitis, or amyloidosis. Before the initiation of canakinumab therapy, the median annual attack number was five (range two to 12), and the most frequently detected mutation was homozygous M694V (n=8 (53%)).

**Figure 2:**
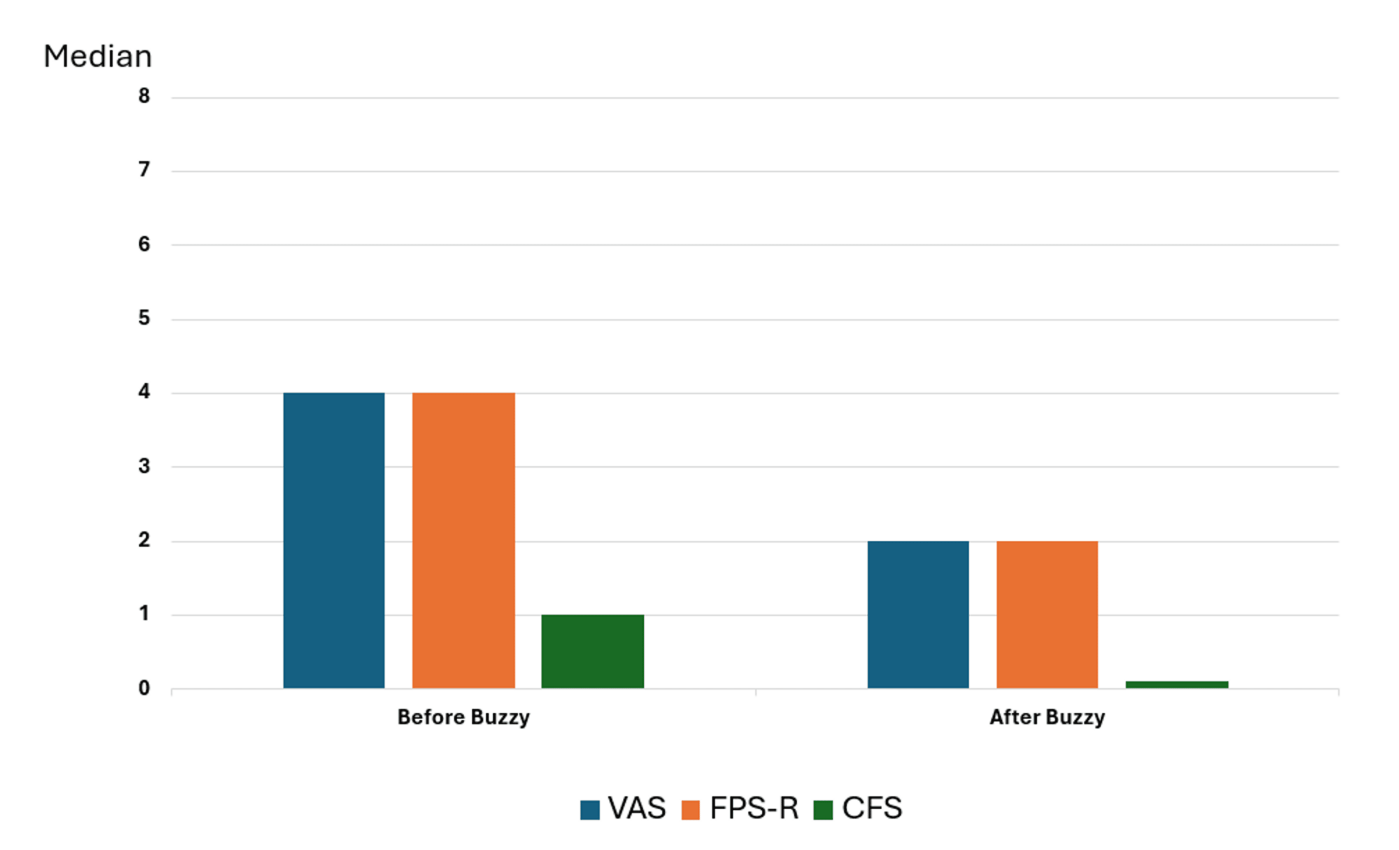
Median VAS, FPS-R, and CFS scores before and after Buzzy® VAS: Visual Analog Scale (VAS); FPS-R: Wong-Baker FACES Pain Rating Scale; CFS: Children’s Fear Scale

Before Buzzy®, the median VAS, FPS-R, and CFS scores were four (range 0 to eight), four (range 0 to eight), and one (range 0 to four), respectively. After Buzzy® application, the median VAS, FPS-R, and CFS scores were two (range 0 to six), two (range 0 to six), and 0 (range 0 to three), respectively. The VAS and FPS-R scores showed a significant reduction (p=0.04 and p=0.008, respectively), indicating a notable decrease in pain. Although the CFS scores also decreased following the use of Buzzy®, this reduction was not statistically significant (p=0.526) (Figure 2).

In patients aged below 12 years, the median VAS, FPS-R, and CFS scores before using Buzzy® were six (range: two to eight), four (range: 0 to eight), and two (range: 0 to four), respectively. After using Buzzy®, the median VAS, FPS-R, and CFS scores were two (range: 0 to six), two (range: 0 to six), and 0 (range: 0 to three), respectively. In patients aged 12 years and above, the median VAS, FPS-R, and CFS scores before using Buzzy® were 0 (range: 0 to two), two (range: 0 to two), and 0 (range: 0 to two), respectively. After using Buzzy®, the median VAS, FPS-R, and CFS scores were 0 (range: 0 to two), two (range: 0 to two), and 0 (range: 0 to 0), respectively. When comparing the VAS, FPS-R, and CFS scores before and after the use of Buzzy® in patients under 12 years of age and those aged 12 years and above, only the pre-treatment VAS scores showed significant differences.

## Discussion

Venipuncture and injections are routine medical procedures for pediatric patients, but they are often perceived as distressing and painful. Therefore, the implementation of effective pain management strategies is imperative. In this study, we demonstrated that the use of Buzzy® significantly reduced pain and anxiety in pediatric patients receiving injections at a rheumatology clinic.

Familial Mediterranean fever is characterized by serosal membrane inflammation, which leads to painful episodes. When patients fail to respond adequately to oral colchicine therapy, it is well-documented that the frequency of attacks can be diminished by administering daily or monthly subcutaneous injections [14,15]. Despite this, pediatric patients often experience considerable anxiety and distress during each injection. The pain associated with injections can induce various psychological effects, including heightened anxiety and phobia, which may exacerbate future pain perceptions, adversely affect treatment adherence, and contribute to therapy avoidance [7,16].

In the literature, the application of Buzzy® has been documented in various clinical procedures, including venipuncture, intramuscular injections, and local dental interventions [11,17]. However, its application in the field of rheumatology remains limited. To date, only one study by Sivri et al. [9] has shown that the use of Buzzy® during venipuncture in a pediatric rheumatology clinic effectively reduces pain and fear. In this study, we assessed the impact of Buzzy® in a pediatric rheumatology clinic, focusing specifically on a patient cohort undergoing subcutaneous canakinumab therapy.

Several studies have explored various methods for reducing pain and anxiety. Distraction techniques, including the use of distraction cards, listening to music, squeezing a ball, and the application of Buzzy®, have been extensively studied. Inal et al. [18] showed that the Buzzy® method was effective in reducing pain during venipuncture in children aged between six and 12 years. Similarly, Canbulat et al. [10] demonstrated that the application of cold and vibration during intravenous catheterization lowers pain and anxiety levels in 176 children aged between seven and 12 years. In a randomized controlled trial by Erdoğan et al. [11], the effects of distraction cards, virtual reality, and the Buzzy® device on pain and anxiety during venipuncture in children aged between seven and 12 years were compared. The Buzzy® group had the lowest VAS, FPS-R, and CFS scores. Additionally, Susam et al. [7] reported in a randomized controlled study involving 64 children aged between three and 10 years that the use of Buzzy® during venipuncture significantly reduced pain and fear while increasing parental satisfaction. Another study evaluated the effect of Buzzy® on pain perception and comfort during local anesthesia in children aged between five and 10 years and found that cold and vibration applied through Buzzy® could reduce pain and anxiety associated with local anesthesia [17]. In a study conducted in our country, the use of the Buzzy® device during measles, mumps, and rubella (MMR) vaccination at 12 months of age in 60 infants led to significantly lower pain scores compared with the control group [19]. Another study conducted in our country, Turkey, demonstrated a significant reduction in pain scores in children who received intramuscular penicillin in the emergency department with the use of Buzzy® [8]. However, in contrast to these findings, some studies reported no significant reduction in pain and anxiety during venous sampling when using the Buzzy® device [20, 21]. In our patient cohort, the VAS and FPS-R scores decreased significantly (p=0.04 and 0.008, respectively). Although the changes in the CFS scores were not statistically significant (p=0.526), they were lower than those recorded before the application of Buzzy®.

As patients age, their pain thresholds may exhibit variability. Furthermore, factors such as gender, previous hospitalization, and previous invasive procedures can influence pain scores in children. Studies on the application of Buzzy® have predominantly focused on children aged between three and 18 years, with a notable preponderance of subjects in the six to 12-year age group [8, 10, 20]. In our study, the ages of the patients ranged from five to 18 years. Among them, five were over 12 years old, while the remaining were under 12. One patient aged 18 years reported not feeling any pain or fear during the injection; therefore, their scores were recorded as 0. When comparing the VAS, FPS-R, and CFS scores before and after the use of Buzzy® in patients aged 12 years and those aged 12 years, only the pre-treatment VAS scores showed significant differences.

In a meta-analysis that included 19 studies investigating the efficacy and safety of Buzzy® in needle-related procedures in children aged 12 years and younger, Buzzy® was found to significantly reduce both pain response and anxiety scores [22]. In future research, it would be beneficial to consider detailed protocol standardization, the use of uniform pain assessment tools, and the inclusion of other potential confounding factors, such as children's psychological status, procedural anxiety, and cultural perceptions of pain. Such methodological improvements could help clarify the underlying causes of heterogeneity, enhance the reliability and generalizability of findings, and contribute to the development of personalized pain management strategies.

The limitations of our study include its single-center design and small sample size.

## Conclusions

In this study, the use of Buzzy® significantly reduced pain in pediatric patients receiving subcutaneous canakinumab for colchicine-resistant FMF, as evidenced by the significant decrease in VAS and FPS-R scores. Although CFS scores also declined, the reduction was not statistically significant. The pain-relieving effect of Buzzy® was more pronounced in patients under 12 years of age. These findings support the effectiveness of Buzzy® as a non-pharmacological pain management tool in pediatric rheumatology patients undergoing repeated injections. However, larger studies with standardized protocols are needed to further validate these results.
